# 4,5-Bis(4-fluoro­phen­yl)-5-hy­droxy-3-(2-methyl­propano­yl)-1-phenyl­pyrrolidin-2-one

**DOI:** 10.1107/S1600536811008786

**Published:** 2011-03-12

**Authors:** Jian-Ying Huang, Fengyan Zhou

**Affiliations:** a Department of Applied Chemistry, Zhejiang Gongshang University, Hangzhou 310035, People’s Republic of China; b Department of Chemistry, Zaozhuang University, Shandong, People’s Republic of China

## Abstract

The title compound, C_26_H_23_F_2_NO_3_, was synthesized by the reaction of 2-(4-fluoro­benzyl­idene)-4-methyl-3-oxo-*N*-phenyl­penta­namide and 4-fluoro­benzaldehyde. The dihedral angles between the mean plane through the pyrrolidine ring (nearly planar; maximum deviation of 0.145 Å for the C atom bearing the hydroxy group) with the phenyl and benzene rings are 37.22 (7), 51.88 (7) and 87.64 (9)°, respectively. The pyyolidine ring is near coplaner, with max offset of 0.145 A for C19 atom.\uff09 In the crystal, mol­ecules are linked by pairs of O—H⋯O hydrogen bonds into inversion dimers, which are further assembled into chains parallel to the *b* axis by weak C—H⋯O hydrogen bonds.

## Related literature

The title compound is an impurity in the preparation of an inter­mediate in the synthesis of atorvastatin {systematic name [*R*-(*R* ^*^,*R* ^*^)]-2-(4-fluoro­phen­yl)-ρ,δ-dihy­droxy-5-(1-methyl­eth­yl)-3-phenyl-4-[(phenyl­amino)­carbon­yl]-1*H*-pyrrole-1-heptenoic acid}, see: Baumann *et al.* (1992 ▶); Sagyam *et al.* (2007 ▶). For the use of atorvastatin as a hypolipidemic and hypochol­esterolemic agent, see: Lea & McTavish, (1997 ▶). For bond-length data, see: Allen *et al.* (1987 ▶).
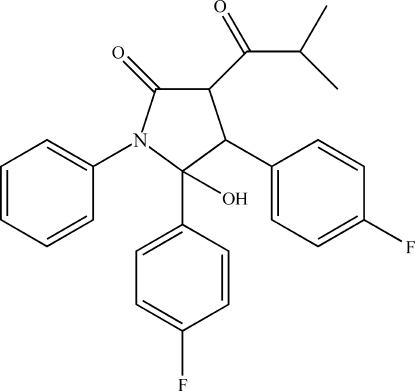


## Experimental

### 

#### Crystal data

C_26_H_23_F_2_NO_3_
*M* *_r_* = 435.45
Monoclinic, 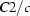

*a* = 24.506 (4) Å
*b* = 10.2180 (15) Å
*c* = 20.554 (3) Å
β = 121.963 (2)°
*V* = 4366.5 (11) Å^3^
*Z* = 8
Mo *K*α radiation
μ = 0.10 mm^−1^
*T* = 153 K
0.15 × 0.15 × 0.10 mm

#### Data collection

Bruker SMART CCD diffractometer
Absorption correction: multi-scan (*SADABS*; Bruker, 2005 ▶) *T*_min_ = 0.986, *T*_max_ = 0.990
10716 measured reflections
4091 independent reflections
2471 reflections with *I* > 2σ(*I*)
*R*_int_ = 0.033

#### Refinement

*R*[*F* ^2^ > 2σ(*F* ^2^)] = 0.039
*wR*(*F* ^2^) = 0.103
*S* = 1.02
4091 reflections
293 parameters
H-atom parameters constrained
Δρ_max_ = 0.15 e Å^−3^
Δρ_min_ = −0.18 e Å^−3^

### 

Data collection: *SMART* (Bruker, 2005 ▶); cell refinement: *SAINT* (Bruker, 2005 ▶); data reduction: *SAINT*; program(s) used to solve structure: *SHELXS97* (Sheldrick, 2008 ▶); program(s) used to refine structure: *SHELXL97* (Sheldrick, 2008 ▶); molecular graphics: *SHELXTL* (Sheldrick, 2008 ▶); software used to prepare material for publication: *SHELXL97*.

## Supplementary Material

Crystal structure: contains datablocks global, I. DOI: 10.1107/S1600536811008786/rz2536sup1.cif

Structure factors: contains datablocks I. DOI: 10.1107/S1600536811008786/rz2536Isup2.hkl

Additional supplementary materials:  crystallographic information; 3D view; checkCIF report

## Figures and Tables

**Table 1:** Hydrogen-bond geometry (Å, °)

*D*—H⋯*A*	*D*—H	H⋯*A*	*D*⋯*A*	*D*—H⋯*A*
C10—H10⋯O1^i^	0.95	2.59	3.489 (3)	157
O2—H2⋯O1^ii^	0.84	1.93	2.7625 (18)	174

Symmetry codes: (i) 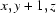; (ii) 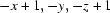.

**Fig. 1.**
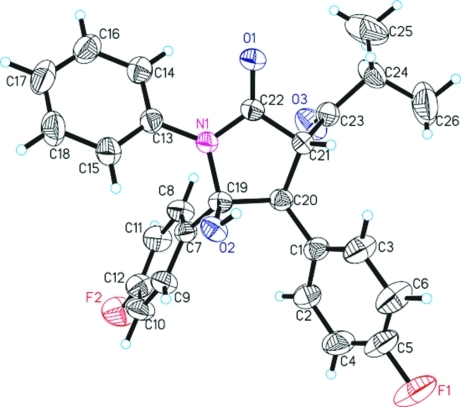
ORTEP view of the title compound. Dispalcement ellipsoids are drawn at 30% probability level.

## supplementary crystallographic information

### Comment

Atorvastatin, {[*R*-(*R*^*^,*R*^*^)]-2-(4-fluorophenyl)-ρ,δ-dihydroxy-5-(1-methylethyl)-3-phenyl-4-[(phenylamino)carbonyl]-1*H*-pyrrole-1-heptenoic acid}, is a selective and competitive inhibitor of the enzyme hydroxyl-methylglutaryl coenzyme-A reductase (HMG-CoA-*R*), which plays a key role in the biosynthesis of cholesterol. By virtue of that activity atorvastatin is useful as a hypolipidemic and hypocholesterolemic agent (Lea & McTavish, 1997). The synthesis of atorvastatin is carried out *via* the critical intermediate 2-[2-(4-fluorophenyl)-2-oxo-1-phenylethyl]-4-methyl-3-oxo-pentanoic acid phenylamide (Sagyam *et al.*, 20077; Baumann *et al.*, 1992). The process for preparing this intermediate is particularly sensitive and vulnerable to the formation of some impurities which may cause product rejection and decreased yields. We report here the crystal structure of one of these impurities.

In the title compound (Fig. 1) bond lengths and angles are within normal ranges (Allen *et al.*, 1987). The five atoms of the pyrrolidine ring are not coplanar, with deviations from the planarity ranging from -0.124 (2) to 0.163 (2) Å. The dihedral angles between the mean plane through the pyrrolidine ring with the C13–C18 phenyl ring and the C1–C6, C7–C12 benzene rings are 37.22 (7), 51.88 (7)° and 87.64 (9)°, respectively. In the crystal structure, the molecules are linked by intermolecular O—H···O hydrogen bonds into dinuclear units (Table 1). The dinuclear units are further assembled into one–dimensional chains along the [010] direction by C–H···O hydrogen bonds.

### Experimental

A mixture of 2-(4-fluorobenzylidene)-4-methyl-3-oxo-*N*-phenylpentanamide (5.10 g, 16.4 mmol), ethyl hydroxyethylmethyl thiazolium bromide (0.60 g, 2.38 mmol), 4-fluorobenzaldehyde (2.16 g, 17.4 mmol) and triethylamine (1.20 g, 11.9 mmol) was heated with stirring to 338 K. The reaction mixture was allowed to stand for 24 h at this temperature. Then 2-propanol (6.0 ml) was added and the mixture was heated to about 373 K. Deionized water (6.0 ml) was dropwise added to the reaction mixture over 30 min while maintaining the temperature at 338 K. After it was gradually cooled to 273 K, a white solid was isolated on a filter and washed with 2-propanol. The solid was recrystallized from ethyl acetate/hexane (1:1 *v*/*v*) and dried under vacuum at 323 K to give the title product as a white solid (2.28 g, yield 32%). Colourless crystals were obtained by vapor diffusion of pentane into an acetone solution over a period of 5 d.

### Refinement

The hydroxy H atom was found in a difference Fourier map and refined using a riding model, with the O—H = 0.84 Å and with *U*_iso_(H) = 1.5 *U*_eq_(O). All other H atoms were placed in geometrically calculated position and refined using a riding model, with C—H = 0.95–1.00 Å and *U*_iso_(H) = 1.2 *U*_eq_(C) or 1.5 *U*_eq_(C) for methyl H atoms.

### Crystal data

**Table:**

C_26_H_23_F_2_NO_3_	*F*(000) = 1824
*M**_r_* = 435.45	*D*_x_ = 1.325 Mg m^−^^3^
Monoclinic, *C*2/*c*	Mo *K*α radiation, λ = 0.71073 Å
Hall symbol: -C 2yc	Cell parameters from 10716 reflections
*a* = 24.506 (4) Å	θ = 2.0–25.6°
*b* = 10.2180 (15) Å	µ = 0.10 mm^−^^1^
*c* = 20.554 (3) Å	*T* = 153 K
β = 121.963 (2)°	Prism, colourless
*V* = 4366.5 (11) Å^3^	0.15 × 0.15 × 0.10 mm
*Z* = 8	

### Data collection

**Table:**

Bruker SMART CCD diffractometer	4091 independent reflections
Radiation source: fine-focus sealed tube	2471 reflections with *I* > 2σ(*I*)
graphite	*R*_int_ = 0.033
φ and ω scans	θ_max_ = 25.6°, θ_min_ = 2.0°
Absorption correction: multi-scan (*SADABS*; Bruker, 2005)	*h* = −29→20
*T*_min_ = 0.986, *T*_max_ = 0.990	*k* = −12→12
10716 measured reflections	*l* = −21→25

### Refinement

**Table:**

Refinement on *F*^2^	Secondary atom site location: difference Fourier map
Least-squares matrix: full	Hydrogen site location: inferred from neighbouring sites
*R*[*F*^2^ > 2σ(*F*^2^)] = 0.039	H-atom parameters constrained
*wR*(*F*^2^) = 0.103	*w* = 1/[σ^2^(*F*_o_^2^) + (0.0294*P*)^2^ + 3.1716*P*] where *P* = (*F*_o_^2^ + 2*F*_c_^2^)/3
*S* = 1.02	(Δ/σ)_max_ < 0.001
4091 reflections	Δρ_max_ = 0.15 e Å^−^^3^
293 parameters	Δρ_min_ = −0.18 e Å^−^^3^
0 restraints	Extinction correction: *SHELXL97* (Sheldrick, 2008), Fc^*^=kFc[1+0.001xFc^2^λ^3^/sin(2θ)]^-1/4^
Primary atom site location: structure-invariant direct methods	Extinction coefficient: 0.00069 (13)

### Special details

**Table:**

Geometry. All e.s.d.'s (except the e.s.d. in the dihedral angle between two l.s. planes) are estimated using the full covariance matrix. The cell e.s.d.'s are taken into account individually in the estimation of e.s.d.'s in distances, angles and torsion angles; correlations between e.s.d.'s in cell parameters are only used when they are defined by crystal symmetry. An approximate (isotropic) treatment of cell e.s.d.'s is used for estimating e.s.d.'s involving l.s. planes.
Refinement. Refinement of *F*^2^ against ALL reflections. The weighted *R*-factor *wR* and goodness of fit *S* are based on *F*^2^, conventional *R*-factors *R* are based on *F*, with *F* set to zero for negative *F*^2^. The threshold expression of *F*^2^ > σ(*F*^2^) is used only for calculating *R*-factors(gt) *etc*. and is not relevant to the choice of reflections for refinement. *R*-factors based on *F*^2^ are statistically about twice as large as those based on *F*, and *R*- factors based on ALL data will be even larger.

### Fractional atomic coordinates and isotropic or equivalent isotropic displacement parameters (Å^2^)

**Table:**

	*x*	*y*	*z*	*U*_iso_*/*U*_eq_	
F1	0.29729 (8)	0.50006 (17)	0.53453 (10)	0.0981 (6)	
F2	0.42759 (8)	0.66971 (13)	0.21676 (8)	0.0818 (5)	
O1	0.48023 (6)	−0.09701 (13)	0.40993 (8)	0.0442 (4)	
O2	0.50669 (6)	0.28658 (13)	0.48691 (7)	0.0412 (4)	
H2	0.5080	0.2276	0.5162	0.062*	
O3	0.32087 (8)	0.03185 (16)	0.26703 (10)	0.0698 (5)	
N1	0.49052 (7)	0.12325 (15)	0.39667 (9)	0.0350 (4)	
C1	0.37194 (9)	0.2815 (2)	0.43135 (12)	0.0413 (5)	
C2	0.35015 (11)	0.4082 (2)	0.40746 (14)	0.0562 (6)	
H2A	0.3519	0.4446	0.3661	0.067*	
C3	0.36879 (12)	0.2328 (2)	0.49159 (13)	0.0608 (7)	
H3	0.3842	0.1469	0.5097	0.073*	
C4	0.32592 (12)	0.4824 (2)	0.44287 (16)	0.0670 (7)	
H4	0.3120	0.5698	0.4269	0.080*	
C5	0.32242 (12)	0.4286 (3)	0.50057 (15)	0.0653 (7)	
C6	0.34365 (13)	0.3059 (3)	0.52658 (15)	0.0721 (8)	
H6	0.3414	0.2708	0.5679	0.086*	
C7	0.46243 (9)	0.35844 (19)	0.35938 (11)	0.0361 (5)	
C8	0.43194 (10)	0.3401 (2)	0.28104 (12)	0.0473 (6)	
H8	0.4186	0.2547	0.2602	0.057*	
C9	0.48343 (10)	0.48238 (19)	0.38900 (12)	0.0443 (5)	
H9	0.5059	0.4958	0.4429	0.053*	
C10	0.47203 (12)	0.5871 (2)	0.34077 (13)	0.0530 (6)	
H10	0.4862	0.6725	0.3610	0.064*	
C11	0.42063 (12)	0.4446 (2)	0.23262 (13)	0.0555 (6)	
H11	0.3997	0.4323	0.1788	0.067*	
C12	0.44041 (12)	0.5657 (2)	0.26446 (13)	0.0530 (6)	
C13	0.54700 (9)	0.11424 (19)	0.39270 (11)	0.0377 (5)	
C14	0.55081 (11)	0.0173 (2)	0.34826 (12)	0.0503 (6)	
H14	0.5164	−0.0429	0.3213	0.060*	
C15	0.59694 (10)	0.2022 (2)	0.43122 (13)	0.0512 (6)	
H15	0.5948	0.2695	0.4617	0.061*	
C16	0.60423 (12)	0.0077 (3)	0.34296 (14)	0.0604 (7)	
H16	0.6066	−0.0597	0.3127	0.072*	
C17	0.65415 (12)	0.0945 (3)	0.38100 (15)	0.0653 (7)	
H17	0.6909	0.0879	0.3770	0.078*	
C18	0.65041 (11)	0.1906 (3)	0.42466 (15)	0.0659 (7)	
H18	0.6850	0.2507	0.4511	0.079*	
C19	0.46645 (9)	0.24691 (18)	0.41043 (11)	0.0357 (5)	
C20	0.39741 (9)	0.20473 (18)	0.39012 (11)	0.0370 (5)	
H20	0.3676	0.2227	0.3342	0.044*	
C21	0.40032 (9)	0.05602 (18)	0.39926 (11)	0.0361 (5)	
H21	0.4023	0.0310	0.4475	0.043*	
C22	0.46109 (9)	0.01616 (19)	0.40310 (10)	0.0348 (5)	
C23	0.34501 (10)	−0.0155 (2)	0.33026 (13)	0.0440 (5)	
C24	0.32304 (11)	−0.1444 (2)	0.34376 (15)	0.0612 (7)	
H24	0.3593	−0.1843	0.3917	0.073*	
C25	0.30332 (16)	−0.2382 (3)	0.27765 (19)	0.1080 (12)	
H25A	0.2696	−0.1979	0.2298	0.162*	
H25B	0.2870	−0.3193	0.2866	0.162*	
H25C	0.3407	−0.2579	0.2738	0.162*	
C26	0.26836 (15)	−0.1156 (4)	0.3559 (2)	0.1135 (13)	
H26C	0.2831	−0.0539	0.3985	0.170*	
H26B	0.2544	−0.1971	0.3680	0.170*	
H26A	0.2323	−0.0772	0.3091	0.170*	

### Atomic displacement parameters (Å^2^)

**Table:**

	*U*^11^	*U*^22^	*U*^33^	*U*^12^	*U*^13^	*U*^23^
F1	0.0963 (12)	0.1070 (13)	0.1038 (12)	0.0287 (10)	0.0616 (11)	−0.0280 (10)
F2	0.1209 (13)	0.0551 (9)	0.0778 (10)	0.0180 (8)	0.0583 (10)	0.0305 (8)
O1	0.0472 (9)	0.0305 (8)	0.0572 (9)	0.0039 (7)	0.0291 (8)	0.0038 (7)
O2	0.0471 (8)	0.0362 (8)	0.0350 (8)	−0.0071 (7)	0.0183 (7)	−0.0017 (6)
O3	0.0736 (12)	0.0648 (11)	0.0495 (10)	−0.0192 (9)	0.0180 (9)	−0.0024 (9)
N1	0.0328 (9)	0.0298 (9)	0.0419 (10)	0.0008 (7)	0.0195 (8)	0.0006 (7)
C1	0.0320 (11)	0.0418 (12)	0.0455 (12)	0.0033 (9)	0.0174 (10)	−0.0048 (10)
C2	0.0540 (15)	0.0462 (14)	0.0736 (16)	0.0108 (11)	0.0374 (14)	0.0034 (12)
C3	0.0721 (17)	0.0615 (16)	0.0571 (15)	0.0233 (13)	0.0398 (14)	0.0070 (12)
C4	0.0627 (17)	0.0484 (15)	0.092 (2)	0.0155 (12)	0.0419 (16)	−0.0056 (14)
C5	0.0519 (16)	0.0776 (19)	0.0657 (17)	0.0131 (14)	0.0307 (14)	−0.0216 (15)
C6	0.0793 (19)	0.086 (2)	0.0597 (16)	0.0261 (16)	0.0425 (15)	0.0023 (15)
C7	0.0393 (11)	0.0329 (11)	0.0389 (11)	0.0028 (9)	0.0226 (10)	0.0010 (9)
C8	0.0578 (14)	0.0382 (12)	0.0428 (13)	−0.0006 (10)	0.0246 (11)	−0.0028 (10)
C9	0.0580 (14)	0.0356 (12)	0.0413 (12)	−0.0037 (10)	0.0277 (11)	−0.0017 (10)
C10	0.0792 (17)	0.0305 (12)	0.0596 (15)	−0.0012 (11)	0.0438 (14)	0.0004 (11)
C11	0.0697 (17)	0.0540 (15)	0.0412 (13)	0.0036 (12)	0.0282 (12)	0.0054 (11)
C12	0.0720 (17)	0.0404 (13)	0.0577 (15)	0.0123 (12)	0.0420 (14)	0.0161 (12)
C13	0.0331 (11)	0.0377 (11)	0.0409 (12)	0.0032 (9)	0.0187 (10)	0.0055 (9)
C14	0.0523 (14)	0.0516 (14)	0.0537 (14)	−0.0020 (11)	0.0327 (12)	−0.0017 (11)
C15	0.0390 (13)	0.0498 (14)	0.0614 (14)	−0.0043 (11)	0.0243 (11)	−0.0029 (11)
C16	0.0642 (17)	0.0649 (16)	0.0687 (16)	0.0097 (14)	0.0465 (14)	0.0043 (13)
C17	0.0474 (16)	0.081 (2)	0.0783 (18)	0.0121 (14)	0.0407 (15)	0.0148 (15)
C18	0.0391 (14)	0.0732 (18)	0.0823 (18)	−0.0084 (12)	0.0300 (14)	0.0022 (15)
C19	0.0372 (11)	0.0321 (11)	0.0353 (11)	0.0002 (9)	0.0176 (9)	−0.0027 (9)
C20	0.0356 (11)	0.0334 (11)	0.0391 (11)	0.0022 (9)	0.0178 (9)	0.0007 (9)
C21	0.0364 (11)	0.0343 (11)	0.0382 (11)	−0.0016 (9)	0.0202 (10)	−0.0005 (9)
C22	0.0351 (11)	0.0316 (11)	0.0344 (11)	−0.0004 (9)	0.0161 (9)	−0.0009 (9)
C23	0.0347 (12)	0.0431 (13)	0.0516 (14)	−0.0005 (10)	0.0210 (11)	−0.0016 (11)
C24	0.0427 (14)	0.0458 (14)	0.0762 (17)	−0.0102 (11)	0.0186 (13)	0.0069 (13)
C25	0.095 (2)	0.0598 (19)	0.122 (3)	−0.0212 (17)	0.025 (2)	−0.0242 (19)
C26	0.081 (2)	0.118 (3)	0.168 (3)	−0.005 (2)	0.083 (3)	0.035 (3)

### Geometric parameters (Å, °)

**Table:**

F1—C5	1.362 (3)	C11—C12	1.363 (3)
F2—C12	1.365 (2)	C11—H11	0.9500
O1—C22	1.228 (2)	C13—C15	1.381 (3)
O2—C19	1.402 (2)	C13—C14	1.384 (3)
O2—H2	0.8400	C14—C16	1.373 (3)
O3—C23	1.208 (2)	C14—H14	0.9500
N1—C22	1.355 (2)	C15—C18	1.392 (3)
N1—C13	1.432 (2)	C15—H15	0.9500
N1—C19	1.484 (2)	C16—C17	1.372 (3)
C1—C3	1.374 (3)	C16—H16	0.9500
C1—C2	1.387 (3)	C17—C18	1.366 (3)
C1—C20	1.511 (3)	C17—H17	0.9500
C2—C4	1.384 (3)	C18—H18	0.9500
C2—H2A	0.9500	C19—C20	1.573 (3)
C3—C6	1.387 (3)	C20—C21	1.528 (3)
C3—H3	0.9500	C20—H20	1.0000
C4—C5	1.351 (3)	C21—C22	1.505 (3)
C4—H4	0.9500	C21—C23	1.532 (3)
C5—C6	1.354 (4)	C21—H21	1.0000
C6—H6	0.9500	C23—C24	1.504 (3)
C7—C9	1.381 (3)	C24—C26	1.516 (4)
C7—C8	1.383 (3)	C24—C25	1.517 (4)
C7—C19	1.517 (3)	C24—H24	1.0000
C8—C11	1.384 (3)	C25—H25A	0.9800
C8—H8	0.9500	C25—H25B	0.9800
C9—C10	1.383 (3)	C25—H25C	0.9800
C9—H9	0.9500	C26—H26C	0.9800
C10—C12	1.350 (3)	C26—H26B	0.9800
C10—H10	0.9500	C26—H26A	0.9800
			
C19—O2—H2	109.5	C17—C16—H16	119.7
C22—N1—C13	122.29 (16)	C14—C16—H16	119.7
C22—N1—C19	112.54 (15)	C18—C17—C16	119.3 (2)
C13—N1—C19	124.01 (15)	C18—C17—H17	120.3
C3—C1—C2	117.6 (2)	C16—C17—H17	120.3
C3—C1—C20	123.64 (19)	C17—C18—C15	121.3 (2)
C2—C1—C20	118.81 (19)	C17—C18—H18	119.4
C4—C2—C1	121.2 (2)	C15—C18—H18	119.4
C4—C2—H2A	119.4	O2—C19—N1	110.20 (15)
C1—C2—H2A	119.4	O2—C19—C7	107.93 (15)
C1—C3—C6	121.6 (2)	N1—C19—C7	113.26 (15)
C1—C3—H3	119.2	O2—C19—C20	113.32 (15)
C6—C3—H3	119.2	N1—C19—C20	101.33 (14)
C5—C4—C2	118.9 (2)	C7—C19—C20	110.82 (16)
C5—C4—H4	120.6	C1—C20—C21	116.98 (16)
C2—C4—H4	120.6	C1—C20—C19	114.49 (16)
C4—C5—C6	122.3 (2)	C21—C20—C19	105.44 (15)
C4—C5—F1	119.2 (3)	C1—C20—H20	106.4
C6—C5—F1	118.5 (3)	C21—C20—H20	106.4
C5—C6—C3	118.5 (2)	C19—C20—H20	106.4
C5—C6—H6	120.7	C22—C21—C20	104.83 (15)
C3—C6—H6	120.7	C22—C21—C23	106.58 (15)
C9—C7—C8	118.89 (18)	C20—C21—C23	113.56 (16)
C9—C7—C19	120.76 (17)	C22—C21—H21	110.5
C8—C7—C19	120.06 (17)	C20—C21—H21	110.5
C7—C8—C11	120.8 (2)	C23—C21—H21	110.5
C7—C8—H8	119.6	O1—C22—N1	125.51 (18)
C11—C8—H8	119.6	O1—C22—C21	124.65 (17)
C7—C9—C10	120.45 (19)	N1—C22—C21	109.84 (16)
C7—C9—H9	119.8	O3—C23—C24	122.0 (2)
C10—C9—H9	119.8	O3—C23—C21	119.63 (19)
C12—C10—C9	118.8 (2)	C24—C23—C21	118.35 (19)
C12—C10—H10	120.6	C23—C24—C26	107.1 (2)
C9—C10—H10	120.6	C23—C24—C25	111.3 (2)
C12—C11—C8	118.0 (2)	C26—C24—C25	112.3 (2)
C12—C11—H11	121.0	C23—C24—H24	108.7
C8—C11—H11	121.0	C26—C24—H24	108.7
C10—C12—C11	123.0 (2)	C25—C24—H24	108.7
C10—C12—F2	118.7 (2)	C24—C25—H25A	109.5
C11—C12—F2	118.3 (2)	C24—C25—H25B	109.5
C15—C13—C14	119.70 (19)	H25A—C25—H25B	109.5
C15—C13—N1	121.04 (18)	C24—C25—H25C	109.5
C14—C13—N1	119.25 (18)	H25A—C25—H25C	109.5
C16—C14—C13	120.3 (2)	H25B—C25—H25C	109.5
C16—C14—H14	119.9	C24—C26—H26C	109.5
C13—C14—H14	119.9	C24—C26—H26B	109.5
C13—C15—C18	118.9 (2)	H26C—C26—H26B	109.5
C13—C15—H15	120.5	C24—C26—H26A	109.5
C18—C15—H15	120.5	H26C—C26—H26A	109.5
C17—C16—C14	120.5 (2)	H26B—C26—H26A	109.5
			
C3—C1—C2—C4	−0.2 (3)	C13—N1—C19—C20	167.56 (16)
C20—C1—C2—C4	179.5 (2)	C9—C7—C19—O2	−13.9 (2)
C2—C1—C3—C6	1.2 (4)	C8—C7—C19—O2	172.30 (17)
C20—C1—C3—C6	−178.5 (2)	C9—C7—C19—N1	−136.22 (19)
C1—C2—C4—C5	−1.4 (4)	C8—C7—C19—N1	50.0 (2)
C2—C4—C5—C6	2.1 (4)	C9—C7—C19—C20	110.7 (2)
C2—C4—C5—F1	−178.5 (2)	C8—C7—C19—C20	−63.1 (2)
C4—C5—C6—C3	−1.1 (4)	C3—C1—C20—C21	19.1 (3)
F1—C5—C6—C3	179.5 (2)	C2—C1—C20—C21	−160.69 (19)
C1—C3—C6—C5	−0.6 (4)	C3—C1—C20—C19	−105.0 (2)
C9—C7—C8—C11	−2.1 (3)	C2—C1—C20—C19	75.2 (2)
C19—C7—C8—C11	171.8 (2)	O2—C19—C20—C1	34.5 (2)
C8—C7—C9—C10	2.3 (3)	N1—C19—C20—C1	152.52 (15)
C19—C7—C9—C10	−171.51 (19)	C7—C19—C20—C1	−87.0 (2)
C7—C9—C10—C12	−0.4 (3)	O2—C19—C20—C21	−95.51 (18)
C7—C8—C11—C12	0.0 (3)	N1—C19—C20—C21	22.51 (18)
C9—C10—C12—C11	−1.9 (4)	C7—C19—C20—C21	142.99 (15)
C9—C10—C12—F2	178.2 (2)	C1—C20—C21—C22	−142.77 (17)
C8—C11—C12—C10	2.1 (4)	C19—C20—C21—C22	−14.23 (19)
C8—C11—C12—F2	−178.0 (2)	C1—C20—C21—C23	101.3 (2)
C22—N1—C13—C15	−139.4 (2)	C19—C20—C21—C23	−130.18 (17)
C19—N1—C13—C15	27.3 (3)	C13—N1—C22—O1	3.7 (3)
C22—N1—C13—C14	41.9 (3)	C19—N1—C22—O1	−164.41 (18)
C19—N1—C13—C14	−151.38 (18)	C13—N1—C22—C21	−175.28 (16)
C15—C13—C14—C16	0.6 (3)	C19—N1—C22—C21	16.6 (2)
N1—C13—C14—C16	179.30 (19)	C20—C21—C22—O1	−179.48 (18)
C14—C13—C15—C18	−0.3 (3)	C23—C21—C22—O1	−58.8 (2)
N1—C13—C15—C18	−179.03 (19)	C20—C21—C22—N1	−0.5 (2)
C13—C14—C16—C17	−0.6 (3)	C23—C21—C22—N1	120.22 (17)
C14—C16—C17—C18	0.3 (4)	C22—C21—C23—O3	−85.3 (2)
C16—C17—C18—C15	−0.1 (4)	C20—C21—C23—O3	29.6 (3)
C13—C15—C18—C17	0.1 (4)	C22—C21—C23—C24	94.0 (2)
C22—N1—C19—O2	95.72 (18)	C20—C21—C23—C24	−151.10 (19)
C13—N1—C19—O2	−72.2 (2)	O3—C23—C24—C26	−88.0 (3)
C22—N1—C19—C7	−143.26 (17)	C21—C23—C24—C26	92.8 (3)
C13—N1—C19—C7	48.8 (2)	O3—C23—C24—C25	35.2 (3)
C22—N1—C19—C20	−24.53 (19)	C21—C23—C24—C25	−144.1 (2)

### Hydrogen-bond geometry (Å, °)

**Table:**

*D*—H···*A*	*D*—H	H···*A*	*D*···*A*	*D*—H···*A*
C10—H10···O1^i^	0.95	2.59	3.489 (3)	157
O2—H2···O1^ii^	0.84	1.93	2.7625 (18)	174

Symmetry codes: (i) *x*, *y*+1, *z*; (ii) −*x*+1, −*y*, −*z*+1.
